# Constipation in adults with neurofibromatosis type 1

**DOI:** 10.1186/s13023-017-0691-4

**Published:** 2017-08-16

**Authors:** Cecilie Ejerskov, Klaus Krogh, John R. Ostergaard, Janne L. Fassov, Annette Haagerup

**Affiliations:** 1 Centre for Rare Diseases, Pediatrics and Adolescent Medicine, Aarhus University Hospital, Palle Juul-Jensens Boulevard 99, 8200 Aarhus N, Denmark; 2 NIDO | Danmark, Research in Education and Health, West Danish Hospital, HEV, Gl. Landevej 61, 7400 Herning, Denmark; 3 Department of Clinical Medicine, Health, Aarhus University, Incuba/Skejby, Palle Juul-Jensens Boulevard 82, 8200 Aarhus N, Denmark; 4 Department of Hepatology and Gastroenterology, Aarhus University Hospital, Noerrebrogade 44 Bygn. 7, 8000 Aarhus C, Denmark

**Keywords:** Neurofibromatosis type 1, Functional gastrointestinal disorders, Self-report questionnaire, Rome criteria, Functional constipation, Irritable bowel syndrome, Functional dyspepsia

## Abstract

**Background:**

Neurofibromatosis type 1 (NF1) is an autosomal-dominant disease characterised by symptoms of the skin, eyes, nervous system and bones. A previous study indicated that constipation, large rectal diameters and prolonged colorectal transit times are common in children with NF1. The aim of the present study was to investigate and compare the prevalence of gastrointestinal symptoms in adult patients with NF1 to their unaffected relatives serving as the control group. Patients with NF1 were recruited from one of two Danish National Centres of Expertise for NF1 and their unaffected relatives were invited to participate as controls. Gastrointestinal symptoms were assessed with a web-based, self-administered, validated, Rome® III diagnostic questionnaire. Logistic regression was used to estimate the prevalence of functional dyspepsia, IBS and functional constipation in each group and the groups were compared using their odds ratios.

**Results:**

The response rates for patients and controls were 66.4% and 82.4%, respectively. We compared 175 patients, median age 34.2 (IQR = 20.1) and 91 of their unaffected relatives, median age 42.0 (IQR = 12). The overall likelihood of fulfilling the diagnostic criteria for functional constipation, irritable bowel syndrome or functional dyspepsia was 33.1% among patients vs. 14.3% among controls, (odds ratio (OR): 2.97; 95% CI: 1.56–5.66) and after adjustment for age and gender (OR: 3.06; 95% CI: 1.62–5.79). The likelihood of functional constipation was higher among patients (OR: 3.80; 95% CI: 1.27–11.31), and this was still true after adjustment (OR: 3.49; 95% CI: 1.14–10.64). The likelihood of irritable bowel syndrome (OR: 2.29; 95% CI: 0.98–5.33) was evident after adjustment (OR: 2.46; 95% CI: 1.10–5.47), whereas there was no difference in the likelihood of functional dyspepsia (OR: 2.35; 95% CI: 0.67–8.32) after adjustment (OR:2.25; 95% CI: 0.70–7.17).

**Conclusions:**

Overall, having symptoms usually attributed to either functional dyspepsia, IBS or functional constipation is more common in adults with NF1 compared to unaffected relatives. Of the three, the likelihood of constipation is markedly higher. The high prevalence of constipation indicates that it is not functional but part of the NF1 disorder.

## Background

Neurofibromatosis type 1 (NF1) is an autosomal dominant disease caused by a mutation in or a deletion of the *neurofibromin* gene on chromosome 17q11.2, causing inactivation of the tumor suppressor gene neurofibromin [1]. The prevalence of NF1 is 1:3000 [2]. Clinical and diagnostic features of NF1 include café au lait macules, cutaneous or plexiform neurofibromas, axillary and inguinal freckling, optic glioma, Lisch nodules and distinctive osseous lesions, such as sphenoid dysplasia or pseudoarthrosis [3]. The NF1 phenotype shows great variability with both intra-family and inter-family expressivity [4]. Thus, some patients have only mild cutaneous manifestations while others have multi-organic involvement. Apart from the physical features, common findings in patients with NF1 are slight mental impairment, learning disabilities and behavioral symptoms [5].

Studies on the gastrointestinal function in patients with NF1 are very rare. In a previous pilot study, we found that children with NF1 had abnormally large rectal diameters and a higher proportion than expected had prolonged colonic transit time [6]. A retrospective assessment of 126 children concluded that an abdominal migraine could be a significant cause of abdominal pain in children with NF1 [7]. For the present study, we hypothesized that adult patients with NF1 have an increased prevalence of gastrointestinal symptoms. The aim was to compare the prevalence of gastrointestinal (GI) symptoms in adults with NF1 and a control group consisting of their relatives without NF1.

## Methods

### Participants

Patients with NF1 were recruited from the outpatient clinic at the Centre for Rare Diseases (CRD), Department of Paediatrics, Aarhus University Hospital, which is one of two Danish National Centres of Expertise for NF1. The centre takes care of all aspects of NF1, regardless of the patient’s age, income and disease severity. Patients were included if they fulfilled the diagnostic criteria of NF1 established by the National Institutes of Health Consensus Conference [3] and were 18 years or older. At the start of the project in December 2015, the 268 patients fulfilling the inclusion criteria were invited to participate between December 2015 and July 2016. The control group comprised relatives without NF1; a partner, a sibling or a parent and who was 18 years or older with no diagnosis of NF1. A total of 125 relatives were invited to participate. The invitations included a link to an online questionnaire and were issued to the eligible patients and their respective relatives during a) the patient’s regular visits to the CRD or b) by letter to the patients who were not scheduled to attend the CRD during the recruitment period. Patients and controls were excluded if they had undergone major abdominal surgery or had known disease affecting their bowel function.

### Protocol

The study was based on the self-administered, validated, Rome® III diagnostic questionnaires comprehensively investigating GI symptoms via the three questionnaire modules; functional dyspepsia, irritable bowel syndrome (IBS) and constipation. It is possible to score and correlate them to the following adult functional GI disorders: functional dyspepsia, IBS, functional constipation, postprandial distress syndrome, epigastric pain syndrome and functional gallbladder, sphincter Oddi disorders and functional defecation disorders [8]. Furthermore, an additional questionnaire described the usage of medicine, exercise, organic GI disease, and major abdominal surgery, the last two being a reason for exclusion. The participants were introduced to the confidentiality procedures and to the use of the questionnaires by the first author. The patients participated on even terms but reading assistance was needed for 17 patients who showed problems with reading or understanding words (*n* = 15), had dyslexia (*n* = 1) or was blind (*n* = 1).

The Rome® questionnaire had been translated from English to Danish accordingly to the guidelines displayed on the Rome Foundation website [9] in the year 2014; a process of six translators active in forward translation, reconciliation, backward translation and finally a consensus meeting was held. Informed consent was collected from patients. Details that might identify the participants have been omitted. The study was approved by the Danish Data Protection Agency (1–16–02-271-15). According to the Ethics Committee of Denmark, no approval was needed for this questionnaire-based investigation.

### Data analysis

We scored the questionnaires accordingly to the Rome III scoring algorithm devised by the Rome Foundation® for functional dyspepsia, postprandial distress syndrome, epigastric pain syndrome, functional gallbladder and sphincter of Oddi disorders, IBS including subtypes, functional constipation, and functional defecation disorders. Study data were collected and managed using REDCap electronic data capture tools hosted at the Department of Clinical Medicine at Aarhus University. REDCap (Research Electronic Data Capture) is a secure, web-based application designed to support data capture for research studies [10].

Statistical analyses were performed with Stata 12 software (StataCorp, College Station, TX). Median and interquartile range of the age were reported in each group and the groups were compared using a permutation test for Mann-Whitney for continuous variables. The variables gender, regular exercise and the usage of laxatives in each group were reported as frequencies and the groups were compared using chi-square test. Logistic regression was used to estimate the prevalence of functional dyspepsia, IBS and functional constipation in patients and controls. The groups were compared using their odds ratios (ORs). The crude prevalence and the crude and adjusted ORs were reported along with their 95% confidence intervals. Specific gastrointestinal symptoms in each group were reported as frequencies and the groups were compared using chi-square test. No corrections were made for the multiple comparisons. Participants from the same family were assumed to be more homogeneous than those from the other family and hence the between-family variation was taken into account in the logistic regression model.

## Results

A total of 178 patients (66.4%) and 103 controls (82.4%) responded. Three participants in the NF1 group were excluded due to inflammatory bowel disease (*n* = 2) or colonic cancer (*n* = 1). Two participants in the control group were excluded due to inflammatory bowel disease (*n* = 2) and ten controls were omitted from the analyses since the information on the family association was lost during the data collection. For basic characteristics on age, gender, exercise and the usage of laxatives on the 175 patients and the 91 controls, see Table 1. When compared to the participating patients the patients who declined to participate were younger, median age 28.2 (IQR = 18.5) (*p* = 0.013) but did not differ in gender (*p* = 0.160). When compared to the participating controls, the relatives who declined to participate did not differ in gender (*p* = 0.095), whereas there was no record of the age of those not responding.

**Table 1:** Basic characteristics of patients and controls

	NF1 patients (*n* = 175)	Controls (*n* = 91)	*p-value*
Age, median (IQR)	34.2 (20.6)	42.0 (12)	0.001
Gender, female, *n* (%)	109 (62%)	41 (45%)	0.007
Regular exercise, *n* (%)	55 (31.4%)	38 (41.8%)	0.094
Usage of laxatives, *n* (%)	8 (4.6%)	5 (5.6%)	0.187

*NF1* neurofibromatosis type 1

### Gastrointestinal symptoms

The proportion of participants fulfilling the criteria for at least one of the three most common functional gastrointestinal disorders (functional dyspepsia, IBS or functional constipation) was 33.1% in the patient group vs. 14.3% the control group, see Table 2.

**Table 2:** The prevalence of gastrointestinal disorders within the groups and compared using the odds ratios

	NF1 patients (*n* = 175)	Controls (*n* = 91)	Odds ratio, *p,* crude	Odds ratio, *p,* adjusted for age and gender
Functional gastrointestinal disorder^a^	33.1% (CI: 26.2–40.1)	14.3% (CI: 7.7–20,8)	2.97 (CI: 1.56–5.66), 0.001	3.06 (CI: 1.62–5.79), 0.001
Functional constipation	14.9% (CI: 9.6–20.1)	4.4% (CI: 0.02–8.6)	3.80 (CI: 1.27–11.31), 0.017	3.49 (CI: 1.14–10.64), 0.028
Irritable bowel syndrom	16.0% (CI: 10.6–21.4)	7.7% (CI: 0.03–12.9)	2.29 (CI: 0.98–5.33), 0.056	2.46 (CI: 1.10–5.47), 0.028
Functional dyspepsia	7.4% (CI: 3.5–11.3)	3.3% (CI: 0–6.9)	2.35 (CI: 0.67–8.32), 0.184	2.25 (CI: 0.70–7.17), 0.170

*NF1* neurofibromatosis type 1, *CI* 95% Confidence Interval, *p p*-value

^a^Any of the three; functional dyspepsia, IBS or functional constipation

*Legend*: The prevalence reported within the group of patients with NF1 and the control group and compared using the odds ratio

### Upper gastrointestinal symptoms

The specific upper gastrointestinal symptoms described in the questionnaire are shown in Table 3. There was no difference in the proportion fulfilling the criteria for functional dyspepsia (patients 7.4% (*n* = 13) and controls 3.3% (*n* = 3)), see Table 2. The proportion fulfilling the criteria for postprandial distress syndrome was 5/175 among patients vs. 1/91 among controls (*p* = 0.360). In both groups, none fulfilled the criteria for epigastric pain syndrome or functional gallbladder and sphincter of Oddi disorders.

**Table 3:** The prevalence for specific symptoms of functional dyspepsia

	NF1 patients (*n* = 175)	Controls (*n* = 91)	*p*
Pain or discomfort in the middle of the chest (not related to heart problems)	13.7% (CI: 9.0–19.7)	6.6% (CI: 2.5–13.8)	0.082
Heartburn	12.6% (CI: 8.0–18.4%)	9.8% (CI: 4.6–17.9)	0.518
Feeling uncomfortably full after a regular sized meal	17.1% (CI: 11.9–23.6)	4.4% (CI: 1.2–10.9)	0.003
Unable to finish a regular size meal	18.3% (CI: 12.9–24.8)	4.4% (CI: 1.2–10.9)	0.002
Pain or burning in the middle of the abdomen	5.7% (CI: 2.8–10.3)	3.3% (CI: 0.6–9.3)	0.386

*NF1* neurofibromatosis type 1, *CI* 95% Confidence Interval, *p p*-value

*Legend*: The prevalence for specific symptoms of functional dyspepsia present at least once per week within the group of patients with NF1 and the control group

### Lower gastrointestinal symptoms

The specific symptoms described in the questionnaire are shown in Tables 4 and 5. The proportion fulfilling the criteria for functional constipation was significant higher in patients (patients 14.9% (*n* = 26) and controls 4.4% (*n* = 4)), see Table 2. The proportion fulfilling the criteria for IBS was in patients 16.0% (*n* = 28) and in controls 7.7% (*n* = 7), see Table 2. There was no difference in IBS subtypes; IBS with constipation (patients 21% (*n* = 6) and controls 14%(*n* = 1) (*p* = 0.260)), IBS with diarrhea (patients 32% (*n* = 9) and controls 57%(*n* = 4), (*p* = 0.790)), mixed IBS (patients 39% (*n* = 11) and controls 14% (*n* = 1), (*p* = 0.055)), and un-subtyped IBS (patients 7% (*n* = 2) and controls 14% (*n* = 1), (*p* = 0.380)). Among the patients and controls with constipation 65% (*n* = 17) and 25% (*n* = 1), (*p* = 0.125), respectively, were identified as cases who would require further investigation to confirm functional defecation disorders.

**Table 4:** The prevalence for specific symptoms of irritable bowel syndrome

	NF1 patients (*n* = 175)	Controls (*n* = 91)	*p*
Discomfort or pain anywhere in the abdomen? ^a^	35.2% (CI: 25.2–39.5)	16.5% (CI: 9.5–25.7)	0.007
Discomfort or pain get better or stop after a bowel movement?	25.1% (CI: 18.9–32.2)	4.3% (CI: 14.0–31.9)	0.567
More frequent bowel movements when discomfort or pain	6.3% (CI: 3.2–11.0)	7.7% (CI: 3.1–15.2)	0.665
Less frequent bowel movements when discomfort or pain	12.7% (CI: 8.0–18.4)	8.9 (CI: 3.9–16.6)	0.355
Looser stools when discomfort or pain	13.1% (CI: 8.5–19.0)	7.7% (CI: 3.1–15.2)	0.183
Harder stools when discomfort or pain	11.4% (CI: 7.1–17.1)	2.2% (CI: 0.3–7.7)	0.010
Often hard or lumpy stools	14.3% (CI: 9.5–20.4)	4.4% (CI: 1.2–10.9)	0.014
Often loose, mushy or watery stools	13.7% (CI: 9.0–19.7)	8.8% (CI: 3.9–16.6)	0.242

*NF1* neurofibromatosis type 1, *CI* 95% Confidence Interval, *p p*-value

^a^At least two to three days a month

*Legend*: The prevalence for specific symptoms of irritable bowel syndrome present at least most of the time within the group of patients with NF1 and the control group

**Table 5:** The prevalence for specific symptoms of functional constipation

	NF1 patients (*n* = 175)	Controls (*n* = 91)	*p*
Fewer than three bowel movements a week	10.9% (CI: 6.7–16.4)	2.2% (CI: 2.7–7.7)	0.013
Hard or lumpy stools	15.4% (CI: 10.4–21.6)	2.2% (CI: 2.7–7.7)	0.001
Straining during bowel movements	17.7% (CI: 12.3–24.2)	5.5% (CI: 1.8–12.4)	0.006
Feeling of incomplete emptying after bowel movements	20.6% (CI: 14.8–27.3)	9.9% (CI: 4.6–17.9)	0.028
Sensation that the stool cannot be passed, (i.e. blocked)	13.1% (CI: 8.5–19.1)	2.2% (CI: 2.7–7.7)	0.004
Press on or around bottom or remove stool in order to complete a bowel movement	4.6% (CI: 2.0–8.8)	0%	-
Difficulty relaxing or letting go during a bowel movement	10.3% (CI: 6.2–17.8)	2.2% (CI: 2.7–7.7)	0.018

*NF1* neurofibromatosis type 1, *CI* 95% Confidence Interval, *p p*-value

*Legend*: The prevalence for specific symptoms of functional constipation present at least often within the group of patients with NF1 and the control group

## Discussion

The present study provides the first systematically collected data on gastrointestinal symptoms among adults with NF1. The patients had a higher prevalence of symptoms usually characterized as functional gastrointestinal disorders. Overall, their likelihood of fulfilling the criteria for functional constipation, IBS, or functional dyspepsia was higher with an OR of three compared to their relatives without NF1. For each of the three conditions functional dyspepsia, IBS and chronic functional constipation, adults with NF1 had a higher likelihood of IBS after adjustment and a markedly higher both crude and adjusted likelihood of functional constipation compared to their relatives.

Functional gastrointestinal disorders are common in the general population. In our study, we explored and assessed the GI symptoms as functional gastrointestinal disorders, which enables us to compare the results to the literature. The prevalence of subjects fulfilling the criteria for functional constipation was 14.9% among adults with NF1 and 4.4% among relatives. In comparable studies using the Rome III criteria, prevalence rates in the general population of 4–7.6% are reported [11–13]. Thus, the occurrence of GI symptoms corresponding to functional constipation seems higher among adults with NF1, both when compared to their relatives and to the general population. In our group of adults with NF1, the prevalence of IBS was 16.0%. This is close to the prevalence found in two other Danish studies using the Rome III criteria in a self-administrated questionnaire to define IBS. One study found a prevalence of 16% among 18–50-year olds and another found a prevalence of 10.5%. Even though the prevalence of functional dyspepsia (7.4%) is close to that in the background population (7.7%) [14], early satiety was more common in adults with NF1 than in controls. This could indicate delayed gastric emptying, either primary or secondary to constipation.

The strengths of our study are that all the invited patients have been assessed and given the diagnosis NF1 by a specialist physician at our center; the use of standardized and validated questionnaires used for collection of data; and the use of relatives as controls by which variations in eating habits as a probable reason for differences should be minimized. Additionally, access to the Danish health system is free which means that patients from all socioeconomic backgrounds representing all NF1 severity grades are followed at our centre. The latter reduces the risk of selection bias.

The study has some limitations. Differences in socioeconomic factors that make eating habits and the intensity of exercise susceptible cannot be excluded nor can unidentified morbidity of the patients. The Rome criteria define functional dyspepsia given that organic disease of the esophagus and the stomach has been excluded with a gastroscopy. Our knowledge of organic upper GI disease is limited to the information from participants given in the questionnaires and the patients’ medical files. The prevalence of functional constipation is greater among women and increases with age [11], and since the proportion of females were higher among patients and the controls were older than the patients we took the differences in age and gender into account in the analysis and reported adjusted ORs.

The present study comprising adults with NF1 was inspired by our previous pilot study among children with NF1 [6]. In that study, children with NF1 had a higher prevalence of constipation, abnormally large diameter of the rectum and a larger proportion than expected had prolonged colorectal transit time. This may indicate that constipation develops early in the life of patients with NF1, and may be a lifelong illness if not addressed. Presumably, patients with NF1 are undertreated since 14.9% of patients were found constipated but only 4.6% patients were using laxatives. A study on gastrointestinal symptoms in children and adolescents with NF1 is awaited and further studies should focus on treatment of constipation and consecutive measurements of constipation characteristics.

The pathophysiology behind a higher prevalence of GI symptoms predominantly correlated to constipation in NF1 is unknown. If constipation develops early in life as indicated by our previous pilot study, we would expect that it is caused by NF1 per se. This makes the term “functional” misleading with respect to bowel symptoms in NF1. It is well established that patients with NF1 often have abnormalities within the nervous system. Abnormalities within the enteric, the peripheral or the central nervous system could cause chronic constipation. Further studies are needed to determine whether constipation in NF1 is mainly associated with prolonged transit through the colon, blunted rectoanal reflexes or by a hyposensate rectum. However, the high prevalence of early satiety found in our group of patients with NF1 could indicate a generalized gastrointestinal motility disorder.

## Conclusion

In conclusion, we found that adults with NF1 have a higher likelihood of having symptoms of constipation (adjusted OR 3.5). This deserves further attention in future studies and in clinical practice. Constipation has consequences for quality of life [15]. Thus, professionals taking care of patients with NF1 should be aware of gastrointestinal symptoms.

## Abbreviations

CRD: Centre for rare diseases
GI: Gastrointestinal
IBS: Irritable bowel syndrome
NF1: Neurofibromatosis type 1
